# Meat Consumption and Risk of Developing Type 2 Diabetes in the SUN Project: A Highly Educated Middle-Class Population

**DOI:** 10.1371/journal.pone.0157990

**Published:** 2016-07-20

**Authors:** A. Mari-Sanchis, A. Gea, F. J. Basterra-Gortari, M. A. Martinez-Gonzalez, J. J. Beunza, M. Bes-Rastrollo

**Affiliations:** 1 Department of Preventive Medicine and Public Health, University of Navarra, Pamplona, Spain; 2 Nutrition Unit–Department of Endocrinology and Nutrition, Hospital de Navarra, Pamplona, Spain; 3 CIBERobn, Instituto de Salud Carlos III, Madrid, Spain; 4 IDISNA, Navarra’s Research Health Institute, Pamplona, Spain; 5 Endocrinology Unit–Department of Internal Medicine, Hospital Reina Sofia, Tudela, Spain; 6 Department of Clinical Sciences, School of Biomedical and Health Sciences, Universidad Europea de Madrid, Laureate International Universities, Madrid, Spain; University of Barcelona, Faculty of Biology, SPAIN

## Abstract

**Background:**

Meat consumption has been consistently associated with the risk of diabetes in different populations. The aim of our study was to investigate the incidence of type 2 diabetes according to baseline total meat consumption in a longitudinal assessment of a middle-aged Mediterranean population.

**Methods:**

We followed 18,527 participants (mean age: 38 years, 61% women) in the SUN Project, an open-enrolment cohort of a highly educated population of middle-class Spanish graduate students. All participants were initially free of diabetes. Diet was assessed at baseline using a semi-quantitative food frequency questionnaire of 136-items previously validated. Incident diabetes was defined according to the American Diabetes Association’s criteria.

**Results:**

We identified 146 incident cases of diabetes after a maximum of 14 years of follow-up period (mean: 8.7 years). In the fully adjusted model, the consumption of ≥3 servings/day of all types of meat was significantly associated with a higher risk of diabetes (HR: 1.85; 95% CI: 1.03–3.31; p for trend = 0.031) in comparison with the reference category (<2 servings/day). When we separated processed from non-processed meat, we observed a non-significant higher risk associated with greater consumption of processed meat and a non-significant lower risk associated with non-processed meat consumption (p for trend = 0.123 and 0.487, respectively). No significant difference was found between the two types of meat (p = 0.594).

**Conclusions:**

Our results suggest that meat consumption, especially processed meat, was associated with a higher risk of developing diabetes in our young Mediterranean cohort.

## Introduction

Type 2 Diabetes Mellitus (T2DM) is a highly prevalent disease worldwide. In 2010, the estimated number of patients with T2DM was 285 million (6.4%), and it was estimated that in 2030 the number of patients will rise to 439 million (7.7%) [1]. It is well known that this metabolic disease leads to increased morbidity and mortality, along with health care costs [2]. Therefore, worldwide efforts are needed to counteract the current diabetes epidemic.

TD2M is a chronic metabolic disease frequently associated with obesity, dyslipidemia, and high blood pressure what is known as metabolic syndrome. Some important risk factors of T2DM are obesity and physical inactivity. However, diet also appears to be an important factor for developing T2DM as well consequential microvascular complications [3,4]. Therefore, diet can play an important role in its prevention [5,6]. In this manner, the Mediterranean dietary pattern has been associated with a lower risk of T2DM in both observational [7] and intervention studies [8,9]. First described in 1960s by Ancel Keys [10], the Mediterranean diet is characterized by its high consumption of fruits, vegetables, whole grains, nuts and legumes, fish, olive oil as the principal source of fat, a high ratio of monounsaturated/saturated fat intake, moderate wine consumption mainly with meals, and low meat consumption.

For a better understanding of the effects of dietary patterns for the prevention of T2DM, researchers have evaluated the impact of isolated food items rather than considering dietary patterns as a whole [11]. One of these items is meat consumption. Meat consumption has been associated with an increase in total as well as cause-specific mortality [12–14].

Since Snowdon and Phillips first reported the positive relation between meat intake and risk of T2DM in a study conducted with a large proportion of vegetarians [15], there have been several cohorts studies [16–23] and a few meta-analysis [24–27] that have evaluated and have confirmed this direct association. However, it would be very interesting to evaluate whether these findings are consistent in the Spanish population more likely prone to follow a Mediterranean dietary pattern.

For this reason, our objective was to evaluate the association between total meat consumption and TD2M in a young Mediterranean cohort, the SUN Project, with a long-term follow-up, up to 14-years.

## Methods

### Study population

We used data from the “Seguimiento Universidad de Navarra” (SUN) Project. The SUN Project is a dynamic, prospective cohort study conducted in Spain that began in 1999 [28]. Participants completed a baseline questionnaire. Additional follow-up questionnaires are mailed every two years. Participants who do not respond the follow-up questionnaires receive up to 5 additional letters reminding them to answer. All participants are university graduates and more than half of them are health professionals living throughout Spain. This provides a wide variation of lifestyles and dietary patterns [29]. Ages range from 20 to 90 years old (median 36). Details of the design and methods of this cohort have been described in detail elsewhere [28].

**Fig 1:**
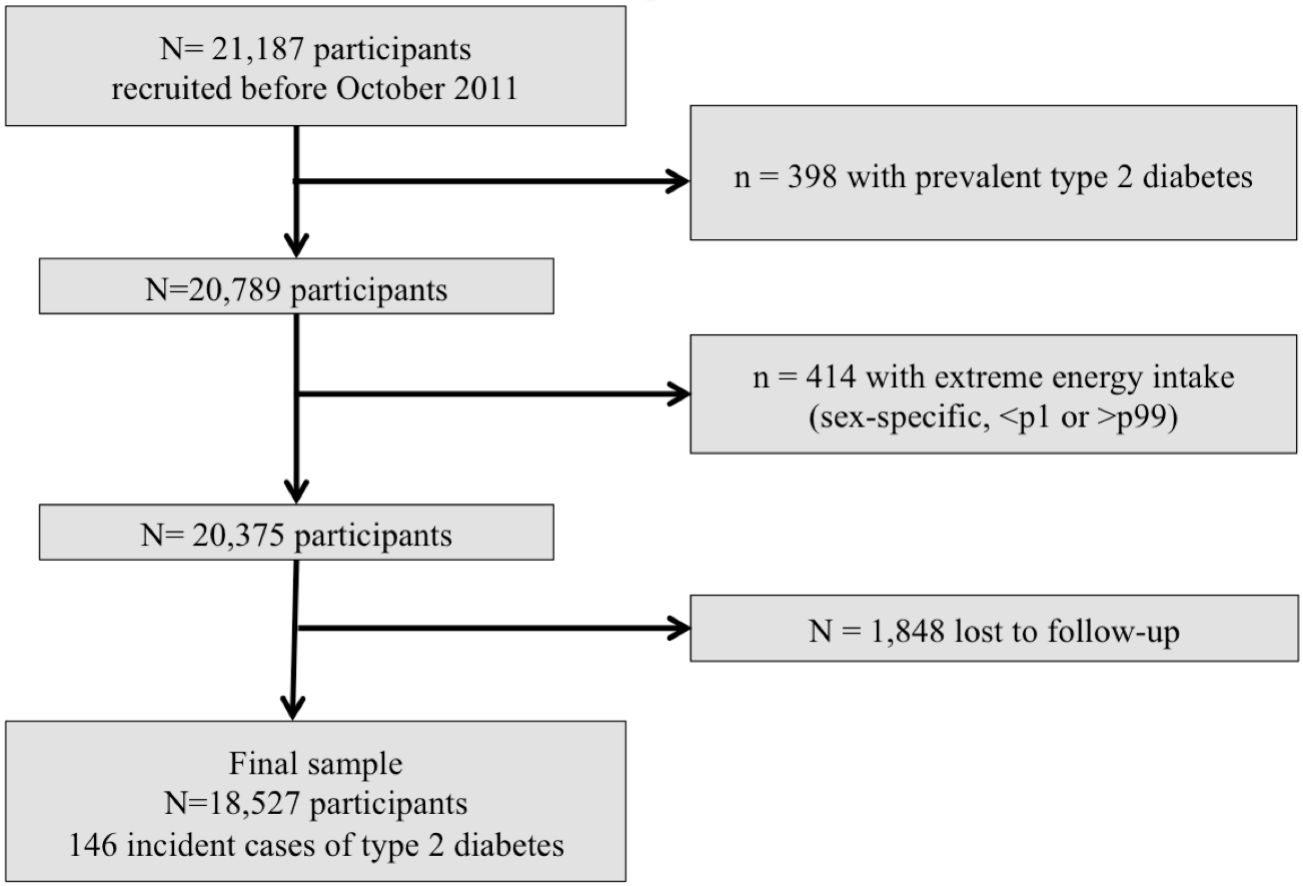
Flow-chart of participants. The SUN Project 1999–2014.

For the present study we assessed 21,187 subjects recruited before October 2011 in order to have enough time for being followed-up for at least during two years. Patients were followed up to a maximum of 14 years The average (SD) follow-up was 8.84 (3.36) years. For the current analysis, we excluded 398 participants who had prevalent diabetes at baseline. We also excluded 414 subjects with an energy intake out of sex-specific predefined limits (below percentile 1 and above percentile 99 of total energy intake). Subjects lost to follow-up without any follow-up questionnaire (n = 1,848) were also excluded. After exclusions, the final sample population included a total of 18,527 participants (Fig 1). The retention in the cohort was 90.9%.

### Exposure assessment

Dietary habits were assessed at baseline using a validated, semi-quantitative food frequency questionnaire with 136-items that has been repeatedly validated in Spain [30–32]. Participants were asked how often they had consumed different types of meat in the previous year. There were nine possible responses ranging from never or seldom, to 6 or more times per day. Each food item was accompanied by the portion size, using data from food composition tables valid for Spain [33]. Total meat consumption was defined as the sum of all types of meat consumed. The correlation coefficient for meat intake in the validation study for this questionnaire was: 0.61 [31].

Two meat groups were defined: 1) “Non-processed meat” that included the consumption of unprocessed (fresh/frozen) beef, pork, veal, lamb, chicken, rabbit, turkey, liver and viscera, and hamburgers (similar to the definition used in previous studies [16,17,25,34]) and 2) processed meat that included bacon, ham, chorizo, salami sausages, pâté, “sobrasada” (a raw cured sausage, typical from Balearic Islands, Spain). Moreover, we defined “non-processed red meat” that included beef, pork, veal, lamb, liver and viscera, and hamburgers. The reproducibility correlation coefficient of processed meat was 0.66 and for red meat was 0.54 [30]. Finally, we categorized daily total meat consumption into three categories: <2, 2-<3 or ≥3 servings/day. A serving contained 100–150 g of meat. In the case of sausages, blood sausages, “sobrasada” and bacon, a serving contained 50 g, and in the case of pâté, a serving contained 25 g. A serving of ham was one slice.

### Outcome assessment

Ascertainment of T2DM in the SUN cohort has been well described before [7]. We considered prevalent cases of diabetes if the participants were treated with either oral antidiabetic agents or insulin or they reported a medical diagnosis at baseline. We considered probable cases of new onset diabetes as those participants who reported a diagnosis of diabetes diagnosed by a doctor in the follow-up questionnaire but did not have diabetes at baseline [7]. These participants were asked to confirm the diagnosis with additional confirmation questionnaires and their medical records were also requested. An endocrinologist, blind to the dietary exposures, confirmed incident cases of T2DM or not, based on the information collected with these questionnaires and the medical records. The incident cases of T2DM were diagnosed using the American Diabetes Association’s criteria [35].

### Covariates assessment

At baseline, information about lifestyle habits (physical activity, tobacco smoking and alcohol intake), medical, socio-demographic and anthropometric variables were recorded. We also collected information about distributions of the major food items in the diet such as carbohydrate, protein and fat intake including monounsaturated fatty acids (MUFA), saturated fatty acids (SFA) and polyunsaturated fatty acids (PUFA) in the latter.

Adherence to the Mediterranean Dietary Pattern (MDP) was evaluated combining 8 items (fruits and nuts, vegetables, fish, legumes, cereals, dairy products, alcohol intake and the ratio MUFA/ SFA) according to the score proposed by Trichopoulou et al [36] while excluding meat and meat products.

The validity of self-reported body mass index (BMI) has been previously validated in a subsample of this cohort. The mean relative error in self-reported weight was 1.45%, and the correlation coefficient between measured and self-reported weight was 0.99 (95% CI: 0.98 to 0.99). For BMI, the mean relative error was 2.64% with a correlation coefficient of 0.94 (95% CI: 0.91–0.97) [37].

To quantify physical activity exerted during leisure time, the time spent performing 17 activities was inquired at baseline. A multiple of resting metabolic rate (MET score) was assigned to each activity, and time spent in each activity was multiplied by the MET score specific for that activity. Total leisure time physical activity was computed as the sum of weekly MET-h of all the activities. In the validation study, weekly MET-h showed to adequately correlate with the objectively measured energy expenditure in a subsample of the cohort (Spearman r = 0.51; 95% CI 0.232–0.707) [38].

### Statistical analysis

The association between total daily meat consumption and the risk of developing T2DM was assessed using Cox regression models. Hazard ratios (HR) and their 95% confidence intervals (95% CI) were calculated using the lowest consumption category as the reference. The first model was adjusted for age and sex. The second one, in addition, included physical activity (MET-h/week), total energy intake (Kcal/day), baseline BMI (kg/m^2^, tertiles), family history of diabetes (none, one or two parents), prevalent hypercholesterolemia and prevalent hypertension. A third model was fitted additionally adjusting for dietary fiber intake (g/day), sugar-sweetened beverages consumption (g/day), smoking status (current, former or never smoker), caffeine intake (mg/day), adherence to Mediterranean dietary pattern (3 categories), prevalent cardiovascular disease, and prevalent cancer. Furthermore, we performed a fourth model that did not include adjustment for BMI.

We additionally fitted a model including 2 separate variables of meat consumption: processed and non-processed meat consumption. To assess differences between the associations for these 2 variables, we performed a χ^2^ Wald test comparing the regression coefficients for the linear terms (servings/day) for each variable. Moreover, we evaluated the effect of meat consumption, red meat, processed meat, and red and processed meat combined with the risk of developing diabetes in grams per day according to tertiles considering the first tertile as the reference category.

We evaluated the interaction of meat consumption according to sex on the development of T2DM using likelihood ratio test comparing the fully adjusted Cox regression model and the same model with the interaction product term.

A number of sensitivity analyses were performed: a) excluding subjects who were following a special diet at baseline, b) excluding subjects out of predefined energy limits <3347,2 kJ/d or >16,736 kJ/d in men and <2092 kJ/d or >14,644 kJ/d in women, c) excluding participants who had 9 or more missing items in the baseline FFQ, and d) excluding participants who had hypertension, hypercholesterolemia, prevalent cancer and prevalent cardiovascular disease.

All P-values were two-tailed and statistical significance was set at the conventional cut-off point of p<0.05. All analyses were performed using STATA 12.0 (StataCorp, College Station, TX).

## Results

The baseline characteristics of the study participants according to the three categories of total meat consumption are shown in Table 1. Those participants who reported higher levels of meat (≥3 servings day) consumption were on average younger (mean age: 35 years), and were more likely to be male and smokers compared with the lowest category of meat consumption. Moreover, participants in the highest category of total meat consumption had the highest total energy, protein and fat intake and the lowest carbohydrate intake. Besides, participants in the lowest meat consumption category were more likely to be female, former smokers and have prevalent cardiovascular disease, cancer, hypercholesterolemia or hypertension at baseline. BMI and adherence to the Mediterranean dietary pattern were fairly balanced between groups.

**Table 1:** Baseline characteristics according to categories of meat consumption (mean and SD, or %). The SUN Project 1999–2014.

*Meat consumption (servings/day)*	*<2*	*2-<3*	*≥3*
*N*	10,461	5,757	2,309
*Age (years)*	40 (12)	36 (11)	35 (11)
*Sex (% female)*	62.6	59.8	57.2
*BMI (kg/m*^*2*^*)*	23.5 (3.5)	23.5 (3.5)	23.5 (3.7)
*Physical activity (MET-h/week)*	22 (22)	21 (22)	22 (27)
*Total energy intake (kJ/day)*	9314 (2769)	11339 (2858)	13790 (3385)
*Macronutrients (% energy)*			
*Carbohydrate intake*	46 (7)	42(6)	39(7)
*Protein intake*	18(3)	18(3)	19(3)
*Fat intake*	35(7)	38(6)	41(6)
*SFA*	12(3)	13(3)	14(3)
*MUFA*	15(4)	16(3)	17(3)
*PUFA*	5(2)	5(2)	6(1)
*Alcohol intake (g/day)*	6(10)	7(11)	8(12)
*Fruit (g/day)*	379(349)	356(311)	390(353)
*Vegetables (g/day)*	527(361)	548(331)	610(418)
*Legumes (g/day)*	22(18)	24(21)	28(23)
*Fish and seafood (g/day)*	96(62)	102(63)	123(109)
*Mediterranean dietary pattern*	3.7 (1.8)	3.8 (1.7)	3.9 (1.7)
*Current smokers (%)*	20.1	22.7	27.0
*Former smokers (%)*	31.1	27.1	24.3
*Prevalent cancer (%)*	4.3	3.0	3.0
*Prevalent cardiovascular disease (%)*	2.6	1.7	1.2
*Prevalent hypercholesterolemia (%)*	18.9	14.5	12.5
*Prevalent hypertension (%)*	7.7	5.9	5.2
*Family history of diabetes (%)*	13.9	13.5	13.6
*Glycemic Index*	52 (5)	52 (4)	52 (5)
*Fiber intake (g/day)*	29 (14)	30 (13)	33 (15)
*Sugar-sweetened beverage intake (g/day)*	55 (111)	75 (139)	103 (175)
*Caffeine intake (mg/day)*	41 (40)	46 (40)	49 (44)

After a median of 8.7 years of follow-up, we identified 146 incident cases of T2DM.

The Cox regression analyses in the overall sample which assessed the association between total meat consumption (categorized in three groups) and incident diabetes are shown in Table 2. After adjustment for several potential confounders, we found that total meat consumption was positively associated with the incidence of T2DM. In the fully adjusted model, the risk of developing T2DM among participants in the highest category of meat consumption (≥ 3 servings/ day) was almost twofold higher (HR 1.85; 95% CI: 1.03–3.31, p<0.031) than in those in the lowest (reference) category (<2 servings/day). Moreover, we conducted a multivariate adjustment not including adjustment for BMI in the models and we observed a higher HR of 1.97 (CI: 1.10–3.55, p<0.020) among participants with meat consumption more than ≥ 3 servings/ day compared with the reference category (<2 servings/day).

**Table 2:** HR and 95% CI for incident type 2 diabetes according to categories of daily meat consumption. The SUN Project 1999–2014.

*Meat consumption (servings/day)*	<2	2-<3	≥3	p for linear trend
Cases/Person-years	88/91,258	41/51,861	17/20,679	
Age and sex-adjusted	1 (Ref.)	1.22 (0.84–1.76)	1.50 (0.90–2.50)	0.089
Multiple-adjusted model ^a^	1 (Ref.)	1.25 (0.85–1.86)	1.65 (0.95–2.87)	0.061
Multiple-adjusted model ^b^	1 (Ref.)	1.33 (0.88–2.00)	1.85 (1.03–3.31)	0.031
Multiple-adjusted model ^c^	1 (Ref.)	1.35 (0.89–2.04)	1.97 (1.10–3.55)	0.020

^a^. Adjusted for age, sex, physical activity (MET-h/week), total energy intake (kJ/day), baseline body mass index (kg/m^2^, tertiles), family history of diabetes (none, one or two parents), prevalent hypercholesterolemia, prevalent hypertension.

^b^. Additionally adjusted for dietary fiber intake (g/day), sugar-sweetened beverages consumption (g/day), smoking status (current, former or never smoker), caffeine intake (mg/day), glycemic index, adherence to Mediterranean dietary pattern (3 categories), prevalent cardiovascular disease, prevalent cancer.

^c^. a+b not including adjustment for BMI

All models are stratified by year of enrollment (2-year periods).

We tested the proportional hazard assumption using the Schoenfeld residuals method. In the fully-adjusted model, hazards of both dummy variables for the second and third categories of total meat consumption were proportional to the hazard of the reference category (p = 0.96 and p = 0.48).

Additionally, we divided the reference category in two categories: less than 1.5 servings/day and the other category from 1.5 to 2 servings/day. When using those participants who eat meat less than 1.5 servings/day as the reference category, we observed in the fully adjusted model a HR of 1.66 (95% CI: 0.90–3.06) (p for trend: 0.116) for those participants who consumed 3 or more servings per day.

**Fig 2:**
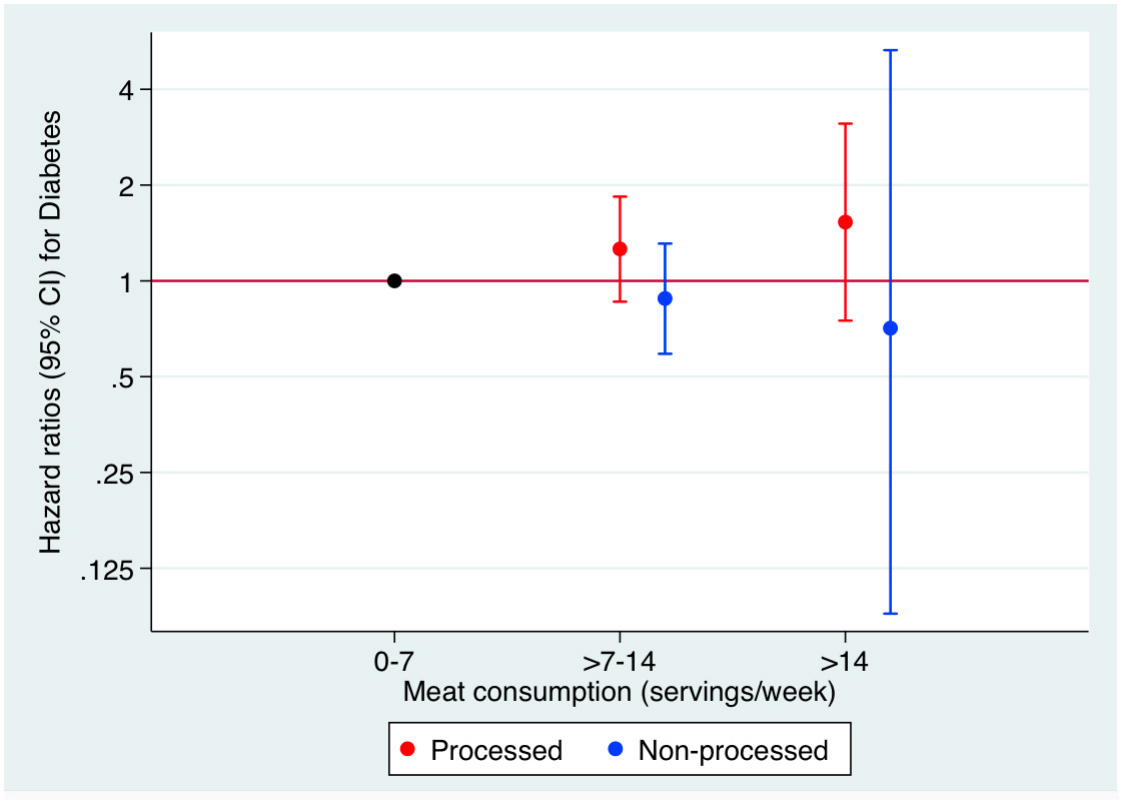
HR for incident type 2 diabetes according to categories of weekly processed and non-processed meat consumption. The SUN Project 1999–2014.

When we assessed adjusting for the consumption of the other type of meat the same model categories of processed and non-processed meat consumption separately, we did not find any significant association (p for trend = 0.123 and 0.487, respectively). Moreover, there were no statistical differences between the linear terms for each variable (p = 0.594). However, results suggested that the association found for meat consumption was mainly due to the consumption of processed meat (Fig 2) (Table 3).

**Table 3:** HR and 95% CI for incident type 2 diabetes according to categories of processed and unprocessed meat consumption. The SUN Project 1999–2014.

*Meat consumption(servings/ week)*	0–7	>7–14	>14	p for trend
**Processed meat**^1^:				
Cases/Persons-years	92/93,188	45/57,477	9/13,133	
Multiple-adjusted model b: HR (95% CI)	1 (Ref.)	1.26(0.86–1.84)	1.53(0.75–3.12)	0.123
Multiple-adjusted model c: HR (95% CI)	1 (Ref.)	1.30(0.88–1.90)	1.60(0.78–3.29)	0.094
Excluding subjects following a special diet at baseline or had hypercholesterolemia, hypertension, CVD and cancer risk				
Cases/Persons-years	21/63,814	19/44,003	6/10,118	
Multiple-adjusted model b: HR (95% CI)	1 (Ref.)	1.87 (1.01–3.46)	3.05 (1.12–8.30)	0.008
**Non-processed meat**^2^:				
Cases/Persons-years	105/106,903	40/54,080	1/2814	
Multiple-adjusted model b: HR (95% CI)	1 (Ref.)	0.88(0.59–1.31)	0.71(0.09–5.31)	0.487
Multiple-adjusted model c: HR (95% CI)	1 (Ref.)	0.92(0.62–1.37)	0.75(0.10–5.65)	0.640
Excluding subjects following a special diet at baseline or had hypercholesterolemia, hypertension, CVD and cancer risk				
Cases/Persons-years	28/77,583	17/40,119	1/2233	
Multiple-adjusted model b: HR (95% CI)	1 (Ref.)	1.07 (0.56–2.02)	1.10 (0.14–8.80)	0.838

^1^Processed meat: included bacon, ham, chorizo, salami sausages, pâté, “sobrasada”

^2^Non-processed meat: that included the consumption of non-processed (fresh/frozen) beef, pork, veal, lamb, chicken, rabbit, turkey, liver and viscera, and hamburgers

b. Adjusted for age, sex, physical activity (MET-h/week), total energy intake (kJ/day), baseline body mass index (kg/m^2^, tertiles), family history of diabetes (none, one or two parents), prevalent hypercholesterolemia, prevalent hypertension, dietary fiber intake (g/day), sugar-sweetened beverages consumption (g/day), smoking status (current, former or never smoker), caffeine intake (mg/day), glycemic index, adherence to Mediterranean dietary pattern (3 categories), prevalent cardiovascular disease, prevalent cancer.

c. b not including adjustment for BMI

We additionally conducted an analysis of meat consumption in grams per day (tertiles) for total meat consumption, non-processed red meat, processed meat and red and processed meat combined as well, in relation to type 2 diabetes. The results are shown in the supplementary Table A in S1 File.

We carried out several sensitivity analyses to evaluate the robustness of our results. The sensitivity analyses excluded subjects who were following a special diet at baseline, the association in the multiple-adjusted model was even stronger than the one in the main analysis (HR 2.45; 95% CI: 1.35–4.45). When we excluded those participants with prevalent hypertension, hypercholesterolemia, cancer and cardiovascular disease the results showed that those participants in the highest category of consumption vs those in the lowest, had a risk of developing T2DM more than 3 times greater (HR 3.31 (95% CI: 1.22–8.94;p <0.001). Other sensitivity analyses showed very similar results to the main analysis (Table 4).

**Table 4:** Sensitivity analyses: HR and 95% CI for incident type 2 diabetes according to categories of daily meat consumption. The SUN Project 1999–2014.

		*Meat consumption (servings/day)*			
*Sensitivity analyses*	Cases/Persons-years	<2	2>3	≥3	p for trend
**Overall**	146/163,798	1 (Ref.)	1.33 (0.88–2.00)	1.85 (1.03–3.31)	0.031
**Excluding participants who followed a special diet at baseline**	130/152,097	1 (Ref.)	1.60 (1.05–2.45)	2.45(1.35–4.45)	0.001
**Energy limits: <3347,2 kJ/d or >16,736 kJ/d in men <2092 kJ/d or >14,644 kJ/d in women**	143/150,995	1 (Ref.)	1.26 (0.82–1.93)	1.88 (1.03–3.42)	0.044
**≥9 missings in FFQ**	95/132,462	1 (Ref.)	1.28 (0.78–2.10)	1.91 (0.97–3.80)	0.062
**Excluding participants who had hypertension, hypercholesterolemia, prevalent cancer and prevalent cardiovascular disease**	48/14,033	1 (Ref.)	2.64 (1.34–5.19)	3.31 (1.22–8.94)	0.001

Adjusted for age, sex, physical activity (MET-h/week), total energy intake (kJ/day), baseline body mass index (kg/m^2^, tertiles), family history of diabetes (none, one or two parents), prevalent hypercholesterolemia, prevalent hypertension, dietary fiber intake (g/day), sugar-sweetened beverages consumption (g/day), smoking status (current, former or never smoker), caffeine intake (mg/day), glycemic index, adherence to Mediterranean dietary pattern (3 categories), prevalent cardiovascular disease, prevalent cancer.

Finally, there was no significant interaction between total meat consumption and sex on the incidence of T2DM (p = 0.29). (Table 5). Although in men, the results of the overall analyses were stronger.

**Table 5:** HR and 95% CI for incident type 2 diabetes according to categories of daily meat consumption according to sex. The SUN Project 1999–2014.

*Meat consumption(servings/day)*	<2	2–3	≥3	*P* for trend
**Men:**				
Cases/Persons-years	68/34,386	29/20,975	15/8,978	
Multiple-adjusted model b: HR (95% CI)	1 (Ref.)	1.22(0.74–2.03)	2.23(1.16–4,28	0.033
**Women:**				
Cases/Persons-years	20/56,872	12/30,855	2/11,701	
Multiple-adjusted model b: HR (95% CI)	1 (Ref.)	1.87(0.95–3.71)	1.08 (0.20–5.94)	0.370

Adjusted for age, sex, physical activity (MET-h/week), total energy intake (kJ/day), baseline body mass index (kg/m^2^, tertiles), family history of diabetes (none, one or two parents), prevalent hypercholesterolemia, prevalent hypertension, dietary fiber intake (g/day), sugar-sweetened beverages consumption (g/day), smoking status (current, former or never smoker), caffeine intake (mg/day), glycemic index, adherence to Mediterranean dietary pattern (3 categories), prevalent cardiovascular disease, prevalent cancer.

## Discussion

In this Mediterranean population of middle-aged Spanish adults with an initially low average body weight, we observed that a higher consumption of meat was associated with a higher risk of T2DM after adjusting for potential confounders. Although the lack of statistical significant, results suggested that this harmful effect was mediated by processed meat. Although, the EPIC (*European Prospective Study on Cancer and Nutrition)* -InterAct study [39], the largest diabetes cohort to date evaluated the effect of meat intake and risk of developing T2DM in eight European countries including Spain, to our knowledge, no previous study has evaluated this association exclusively in a young Mediterranean population.

Our results are in agreement with previous published results. Four meta-analyses of cohort studies previously demonstrated the positive association between total meat consumption and diabetes development [24–27].

In relation to the total meat consumption and risk of diabetes, an increased risk was observed in the meta-analysis conducted by Micha et al [26] in 2010. Each serving per day of total meat was associated with 12% (RR 1.12; 95% CI: 1.05–1.19) higher risk of diabetes mellitus. Nevertheless, in the study by Aune’s et al [24] in 2009 that included 5 cohorts studies, no clear association between meat intake and diabetes risk was found (RR 1.17; 95%CI: 0.92–1.48). However, after excluding the Chinese study, one of the five cohort studies, this association emerged statistically significant (RR 1.31; 95% CI: 1.12–1.52). In this article by Villegas et al [40], conducted in a sample of Chinese women, the reported findings are not consistent with the others studies included in this meta-analysis (RR 0.82; 95%CI: 0.69–0.98). It showed a decreased risk of development diabetes with higher meat intake instead of an increased risk. It may, perhaps, be due to lower consumption of processed meat in this Chinese cohort, compared with other cohorts studies [17,41]. A direct relationship has also been observed in prospective studies that were not included in the above-mentioned meta-analyses [39,42–44]. In the study of Feskens et al. [27], the most recent meta-analysis, conducted in 2013, they reported a RR of 1.15 (95% CI: 1.07–1.24) per 100 g in total meat intake.

Regarding processed meat consumption, an increase of 50 g/day was associated with a higher risk of diabetes in Aune et al. [24]: 1.57 (95% CI: 1.28–1.93) and 1.32 (95% CI: 1.19–1.48) in Feskens et al. [27]. In Micha et al. [26] they observed a RR of 1.19 (95% CI: 1.11–1.27) for each additional serving of processed meat, and in Pan et al. [25] they found a RR of 1.51 (95% CI: 1.25–1.83).

Recently, in a meta-analysis published by Fretts et al. they observed that meat consumption was associated with higher glucose and insulin concentrations in non-diabetic Caucasians and that this effect was not modified by genes related to glucose or insulin [45]. This study is consistent with the evidence that meat consumption affect glucose metabolism and increases risk of developing diabetes.

Our results were attenuated after adjustment for BMI, maybe due to the independent effect of meat consumption on both adiposity [46] and development of T2DM. Therefore, BMI may explain part of the elevated risk of meat consumption on T2DM.

Moreover, we observed an effect modification by sex, although not statistically significant. In men, the results of the overall analyses were stronger, while in women we did not observe this positive association, perhaps due to the scarcity of cases of T2DM in women compared to men (34 vs 112).

Our findings are in line with these studies in relation to the total consumption of meat and diabetes risk. It is interesting to notice that our results are consistent with those performed in populations with such different baseline characteristics: age, sex (e.g. participants in the EPIC cohort had a mean age of 52 years, the Women’s Health Study aged ≥45 years, while the average age of our cohort is 38 years), in different geographic locations, with different dietary patterns and meat consumption and with different follow-up of participants. This all emphasizes the fact that meat consumption can increase the risk of diabetes development.

In addition, previous results from our cohort showed that a higher consumption of fast food, defined as sausages, hamburgers and pizza, was associated with a higher risk of gestational diabetes [47].

Although the exact mechanism by which meat consumption may increase the risk of T2DM is not clear, some plausible hypothesis have been proposed. A recent article published by Kim et al [48] reviewed in detail the metabolic etiologies through which meat may be related to the development of T2DM. Processed meat is preserved for long term and contains salt or other preservatives such as nitrites. The content of salt per gram of product is, on average, 400% higher in processed meat than in unprocessed meat [49]. Those preservatives are harmful for pancreatic beta cells. In fact, the use of nitrites play an important role in pancreatic dysfunction by the formation of nitrosamines that can cause T2DM [50]. Moreover, the meat content of saturated and trans-fatty acids affects insulin sensitivity [51]. It is known that there is an association between animal fat and saturated fat intake with hyperinsulinemia and insulin resistance [52–53]. Moreover, the content of saturated fat can produce obesity, a known risk factor of T2DM [54–55]. Also, meat is an important source of dietary cholesterol which has been defined as a risk factor of developing T2DM [56]. The presence of advanced glycation end-products in meat as a result of cooking or processing meat, produced an increased risk of T2DM in animals and in humans [57–58].

It is known that meat is an excellent source of highly bioavailable (30–60%) heme iron. Iron intake and iron storage, shown by increased ferritin concentration, have been described to have a positive association with a higher risk of developing diabetes [59]. Recently, this association has been described between high heme iron dietary intake and the risk of new-onset diabetes in a Mediterranean population, such as in our study cohort, although the average age of the population study was different [60]. Moreover, the pro-oxidant effect of iron and its capacity to produce hydroxyl radicals as a possible cause of pancreatic dysfunction has been proposed [61]. It seems that the increased risk for developing T2DM may be due to the interactions between different components of meat, namely, saturated fat, salt and nitrates, iron, advanced glycation end products and trimethylamine N-oxide, a key molecule derived from dietary carnitine and coline mediating the risk of T2DM [48].

Some of the non-significant results might be alternatively explained by the fact that metabolic syndrome and obese patients excrete more nitrite and nitrate due to a problem of nitrogen disposal [62] than the source from meat consumption. Therefore, it might be thought that obesity is more important than consuming higher proportions of meat, although we have adjusted our results for baseline BMI.

In our study, participants belonging to the higher meat consumption group were younger and more likely to be smokers compared with participants in the lowest meat consumption category. It seems Spanish young people are adopting unhealthy lifesytle. In fact, our research group has already found evidence of the abandonment of Mediterranean diet and the adoption of the “Western diet” in the Spanish population, especially among young people, smokers and sedentary participants [63,29].

Therefore, it is important to identify and promote strategies based on current scientific evidence in order to stop the increasing trend of this epidemic disease, such as maintaining and promoting our traditional Mediterranean diet. It is a public health priority to reduce the amount of preservatives, salt and the consumption of total meat (especially processed meat), and to optimize the diet of young and elderly people as well as their lifestyle behavior.

Our study has some limitations. The use of a validated FFQ to evaluate meat consumption provides us only subjective information and we cannot rule out the existence of information bias. However, we used a FFQ previously validated in a Spanish population [30–32] with a reasonable correlation coefficient for our main exposure. Since the possible measurement error in the assessment of diet is expected to be non-differential, it would bias our results toward the null value. Moreover, we did not consider the type of cooked meat, which could increase the risk of developing type 2 diabetes. Another limitation is the absence of repeated measurements of diet during our follow up. Consequently, it is not possible to exclude an information bias, because our participants may have changed their meat consumption during follow-up.

Another potential limitation of our study is the underreport of self-reported diabetes diagnoses. However, all participants of the SUN cohort are university graduates, highly educated, highly motivated and more than half of them are health professionals, therefore we would expect it improbable to have misclassification of both exposure and outcome. Moreover, we must acknowledge the possibility of a lack of statistical power to separately assess the effect of different types of meat on the risk of diabetes.

Finally, another potential disadvantage of our study is that the sample is not representative of the general population, because our population is composed by middle aged people with a high level of education. We should be cautious towards extrapolating of our results to the general population. Therefore, the generalizability of our results must be based on the possible biological mechanisms involved and not on statistical representativeness of the sample. By contrast, this limitation increased the internal validity of our results because of the high level of education and homogeneity of our cohort (all participants are university graduates) which reduces the potential confounding related to socioeconomic status.

The major strengths of the current study include its prospective design, its relatively large sample size and long-term follow-up, and its high retention rate. All these would potentially minimize the possibility of selection or recall bias. Another important strength is the use of a validated FFQ to assess meat consumption, and the medical confirmation of diabetes diagnoses, that would lead to a high specificity. Moreover, we adjusted the models for a wide array of potential confounders, so we consider residual confounding unlikely, although still possible. The sensitivity analyses carried out in our study also confirm the robustness of our findings.

## Conclusions

In conclusion, our data suggest that a higher meat consumption was associated with elevated risk of T2DM in our young Spanish cohort. Future studies in other Mediterranean populations are needed to confirm our findings specially differentiating processed and non-processed meat.

## Supporting Information

S1 File Table A. HR and 95% CI for incident type 2 diabetes according to types of meat consumption. in grams per day (tertiles). The SUN Project 1999–2014. Table B. HR and 95% CI for incident type 2 diabetes according to meat food products consumption. The SUN Project 1999–2014.
(DOCX)
