# Supplementary material for: Meat Consumption and Risk of Developing Type 2 Diabetes in the SUN Project: A Highly Educated Middle-Class Population
Source: PLoS One. 2016 Jul 20;11(7):e0157990. doi: 10.1371/journal.pone.0157990 (PMC4954662; doi:10.1371/journal.pone.0157990)
Supplement: S1 File — Table A. HR and 95% CI for incident type 2 diabetes according to types of meat consumption. in grams per day (tertiles). The SUN Project 1999–2014. Table B. HR and 95% CI for incident type 2 diabetes according to meat food products consumption. The SUN Project 1999–2014. (DOCX) [file pone.0157990.s001.docx]

**Supporting Information**

**S1 Table. HR and 95% CI for incident type 2 diabetes according to types of meat consumption in grams per day (tertiles). The SUN Project 1999-2014.**

| ***Red meat (grams/day)*** | **T1** | **T2** | **T3** | ***P* for trend** |
| --- | --- | --- | --- | --- |
| Cases/Persons-years | 63/63,465 | 45/54,235 | 38/46,098 |  |
| Age and sex-adjusted | 1 (Ref.) | 0.79(0.54-1.16) | 0.99(0.66-1.49) | 0.933 |
| Multiple-adjusted model a HR (95% CI) | 1 (Ref.) | 0.82(0.56-1.21) | 0.97(0.63-1.49) | 0.944 |
| Multiple-adjusted model b HR (95% CI) | 1 (Ref.) | 0.83(0.57-1.23) | 0.95(0.62-1.48) | 0.882 |
| ***Meat consumption (grams/day)*** | **T1** | **T2** | **T3** | ***P* for trend** |
| Cases/Persons-years | 65/57,045 | 40/54,214 | 41/52,539 |  |
| Age and sex-adjusted | 1 (Ref.) | 0.87(0.59-1.30) | 1.21(0.81-1.79) | 0.414 |
| Multiple-adjusted model a HR (95% CI) | 1 (Ref.) | 0.89(0.59-1.34) | 1.27(0.82-1.95) | 0.321 |
| Multiple-adjusted model b HR (95% CI) | 1 (Ref.) | 0.92(0.61-1.38) | 1.36 (0.86-2.15) | 0.226 |
| ***Processed meat (grams/day)*** | **T1** | **T2** | **T3** | ***P* for trend** |
| Cases/Persons-years | 63/54,709 | 45/58,291 | 38/50,798 |  |
| Age and sex-adjusted | 1 (Ref.) | 0.93(0.63-1.36) | 1.16(0.77-1.73) | 0.278 |
| Multiple-adjusted model a HR (95% CI) | 1 (Ref.) | 0.88(0.60-1.29) | 1.17(0.76-1.79) | 0.189 |
| Multiple-adjusted model b  HR (95% CI) | 1 (Ref.) | 0.90 (0.61-1.33) | 1.22(0.78-1.89) | 0.131 |
| ***Processed meat and red meat (grams/day)*** | **T1** | **T2** | **T3** | ***P* for trend** |
| Cases/Persons-years | 63/56,426 | 38/54,143 | 45/53,229 |  |
| Age and sex-adjusted | 1 (Ref.) | 0.81(0.54-1.21) | 1.24(0.85-1.83) | 0.340 |
| Multiple-adjusted model a HR (95% CI) | 1 (Ref.) | 0.81(0.54-1.22) | 1.26(0.83-1.91) | 0.347 |
| Multiple-adjusted model b HR (95% CI) | 1 (Ref.) | 0.83 (0.55-1.24) | 1.31(0.84-2.05) | 0.288 |

a. Adjusted for age, sex, physical activity (MET-h/week), total energy intake (kJ/day), baseline body mass index (kg/m^2^, tertiles), family history of diabetes (none, one or two parents), prevalent hypercholesterolemia, prevalent hypertension.

b. Additionally adjusted for dietary fiber intake (g/day), sugar-sweetened beverages consumption (g/day), smoking status (current, former or never smoker), caffeine intake (mg/day), glycemic index, adherence to Mediterranean dietary pattern (3 categories), prevalent cardiovascular disease, prevalent cancer.

All models are stratified by year of enrollment (2-year periods).

**S2 Table. HR and 95% CI for incident type 2 diabetes according to meat food products consumption. The SUN Project 1999-2014.**

| ***Hamburger*** | **Low consumption (p<50)** | **High consumption (p≥50)** |
| --- | --- | --- |
| Cases/Persons-years | 85/81,061 | 49/77,789 |
| Age and sex adjusted : HR (95% CI) | 1 (Ref.) | 1.11 (0.76-1.62) |
| Multiple-adjusted model a: HR (95% CI) | 1 (Ref.) | 1.05 (0.71-1.54) |
| Multiple-adjusted model b: HR (95% CI) | 1 (Ref.) | 1.04 (0.71-1.53) |
| ***Sausages*** | **Low consumption (p<50)** | **High consumption (p≥50)** |
| Cases/Persons-years | 105/118,330 | 31/40,866 |
| Age and sex adjusted: HR (95% CI) | 1 (Ref.) | 1.30 (0.87-1.93) |
| Multiple-adjusted model a: HR (95% CI) | 1 (Ref.) | 1.30 (0.86-1.96) |
| Multiple-adjusted model b: HR (95% CI) | 1 (Ref.) | 1.30 (0.85-1.98) |
| ***Bacon*** | **Low consumption**  **(p<50)** | **High consumption**  **(p≥50)** |
| Cases/Persons-years | 83/82,751 | 54/73,6 |
| Age and sex adjusted : HR (95% CI) | 1 (Ref.) | 0.98 (0.70-1.38) |
| Multiple-adjusted model a: HR (95% CI) | 1 (Ref.) | 1.07 (0.76-1.50) |
| Multiple-adjusted model b: HR (95% CI) | 1 (Ref.) | 1.03 (0.73-1.46) |
| ***Liver*** | **Low consumption**  **(p<50)** | **High consumption**  **(p≥50)** |
| Cases/Persons-years | 112/128,895 | 20/30,564 |
| Age and sex adjusted: HR (95% CI) | 1 (Ref.) | 0.94(0.58-1.52) |
| Multiple-adjusted model a: HR (95% CI) | 1 (Ref.) | 0.91 (0.57-1.45) |
| Multiple-adjusted model b: HR (95% CI) | 1 (Ref.) | 0.93 (0.58-1.49) |
